# Volatile Organic Compounds in Teas: Identification, Extraction, Analysis, and Application of Tea Aroma

**DOI:** 10.3390/foods14152574

**Published:** 2025-07-23

**Authors:** Qin Zeng, Huifeng Wang, Jiaojiao Tuo, Yumeng Ding, Hongli Cao, Chuan Yue

**Affiliations:** 1 Integrative Science Center of Germplasm Creation in Western China (CHONGQING) Science City, College of Food Science, Southwest University, Chongqing 400715, China; zerlindarey@163.com (Q.Z.); 19562247190@139.com (H.W.); 18875503571@163.com (J.T.); ying731@swu.edu.cn (Y.D.); 2 Chongqing Key Laboratory of Speciality Food Co-Built by Sichuan and Chongqing, Southwest University, Chongqing 400715, China

**Keywords:** tea aroma, tea classification, VOCs identification, VOCs extraction and analysis, aroma applications

## Abstract

Volatile organic compounds (VOCs) are important for teas’ quality and act as a critical evaluative criterion in teas. The distinctive aromatic profile of tea not only facilitates tea classification but also has potential applications in aroma-driven product innovation. In this review, we summarized the tea aroma from tea classification, VOCs extraction methodologies, and VOCs detection techniques. Moreover, the potential utilization of tea aroma in the future, such as applications in essential oil refinement, food flavor enhancement, and functional fragrance for personal health care, was proposed. Our review will provide a solid foundation for further investigations in tea aroma and offer significant insights into the development and application of tea fragrance.

## 1. Introduction

As one of the most ancient and widely consumed beverages globally, tea holds profound cultural significance and immense commercial importance. Botanically, it is defined as a processed product derived from the fresh leaves of *Camellia sinensis* [*Camellia sinensis* (L.) O. Kuntze] or *Camellia assamica* [*Camellia sinensis* var. *assamica* (Mast.) Kitamura]. Tea quality is determined by multiple factors, including plant genetics, agroecological conditions, picking standards, and postharvest processing [1,2,3]. The aromatic profile of tea, which serves as a critical quality indicator, provides an objective basis for evaluating product grades and processing precision. Furthermore, the aromatic profile of tea determines its olfactory characteristics, which play a decisive role in shaping consumer preferences [4]. However, the compounds and their composition in tea aroma are complex remaining to be revealed. Therefore, systematically identifying and accurately classifying VOCs and understanding their biosynthesis pathways are essential to tea science research. And it is a significant implication for enhancing quality control and guiding market-driven product development.

Beyond their role in tea quality, VOCs also have significant potential as natural fragrance ingredients for flavor enhancement and scent formulation in the food, beverage, and cosmetic industries. For instance, essential oils derived from tea can effectively mask undesirable odors from fatty acids, oils, and surfactants in cosmetic products, while key constituents such as linalool, geraniol, and limonene impart favorable aromas [5]. However, practical applications face substantial challenges due to the inherent complexity of VOCs, including their trace concentrations, high volatility, and susceptibility to environmental factors such as light, heat, and humidity [6,7]. This is exemplified by the thermally induced unpleasant odor in green tea beverages during pasteurization [8], which emphasizes the need for refinements in extraction methodologies and systematic investigations into aroma compounds’ stability and enantiomeric effects to maximize industrial utility. Thus, there is a significant challenge in VOCs identification, extraction, and utilization presently.

Driven by the expanding tea market and intensifying consumer demand for quality, methodologies for investigating tea aroma characteristics have evolved significantly. From initial standalone sensory evaluations to their integration with modern instrumental analyses, these advancements have substantially enhanced our ability to characterize tea aroma profiles. Tea contains a complex array of VOCs, including aldehydes, ketones, esters, acids, alcohols, hydrocarbons, phenols, pyrazines, and furans, with compositional variations directly determining aromatic properties [9,10]. To date, hundreds of volatile compounds have been detected in teas. However, the knowledge of the aroma of tea is only the tip of the iceberg.

The variation in composition, concentration, and quantitative ratios of VOCs distinctly determines aromatic profiles and perceived intensity, enabling the establishment of aroma fingerprints for tea identification and authentication. Trained tea tasters can assess product quality, classify varieties, and identify processing flaws by analyzing these volatile varieties. However, human olfaction faces inherent limitations in precisely discerning specific aromatic compounds associated with particular sensory attributes, especially for volatiles with similar structures. Current analytical technologies also exhibit notable constraints, including inadequate sensitivity and resolution, which hinder the detection of trace compounds with low olfactory thresholds. Consequently, both sensory evaluation and instrumental detection methods require further advancement.

Existing reviews predominantly focus on the identification and classification of VOCs, while often lack thorough reports on extraction methodologies, detection techniques, and application prospects. In contrast, this review not only focuses on the characteristic VOCs and their contributions to the flavor profiles of six major tea categories but also systematically evaluates recent advancements in extraction and identification methodologies. We identify key limitations of current approaches and propose targeted strategies for methodological refinement. Furthermore, by integrating a holistic framework spanning classification, extraction, analysis, and application domains, this review bridges fundamental research and methodological innovations with industrial applications. In terms of applications, this review emphasizes emerging uses in essential oils, food additives, daily necessities, and especially in health care, providing a scientific framework for innovative utilization and development of tea aroma.

## 2. Different Tea Types Have Different Flavors

### 2.1. Tea Types Classification

**Figure 1:**
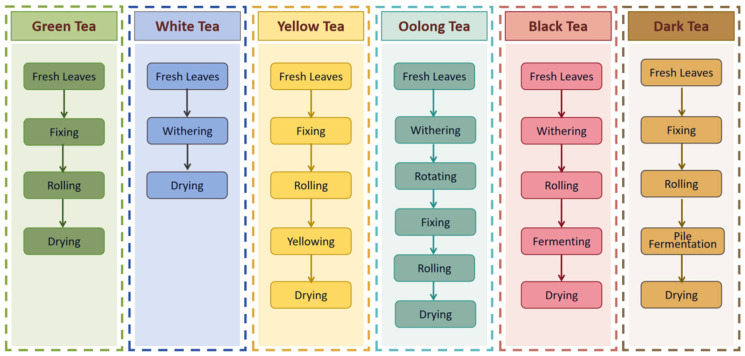
Classification of the six major tea types and processing methods.

Teas are commonly classified into six main categories: green tea, white tea, yellow tea, oolong tea, black tea, and dark tea, based on their distinct processing methods, chemical composition, and sensory characteristics (Figure 1).

Green tea, rich in polyphenols and catechins, is renowned for its antioxidant, anti-cancer, antiviral, and hypoglycemic properties [11]. As a non-fermented tea, it undergoes three primary processing stages: fixing, rolling, and drying. Immediate fixation after harvesting inhibits enzymatic oxidation, thereby preserving chlorophyll content and native phytochemicals. Representative varieties include Xihu Longjing, Biluochun, and Xinyang Maojian, which exemplify China’s premium teas. White tea, categorized as lightly fermented tea, undergoes minimal processing through withering and drying, which helps retain its natural aroma compounds and high antioxidant levels. Studies suggest potential health benefits associated with white tea, including possible anti-inflammatory, anti-mutagenic, anti-cancer, and neuroprotective properties [12]. However, claims regarding anti-cancer and anti-tumor effects require further substantiation. White teas are typically classified into four grades, including Baihao Yinzhen, Bai Mudan, Gongmei, and Shoumei, which are primarily based on the maturity of the buds and leaves. Yellow tea, a partially fermented category, undergoes a unique process of fixing, rolling, yellowing, and drying. This process leads to its signature characteristics: yellow-tinged leaves and golden liquor with a distinctive aroma. Notable varieties include Mengding Huangya, Junshan Yinzhen, and Huoshan Huangya. Oolong tea, characterized as semi-fermentation, undergoes a sequential process involving withering, rotating, fixing, rolling, and drying. Occupying an intermediate position between green and black teas, oolongs are renowned for their complex and nuanced flavor profiles. Tieguanyin, Dahongpao, and Fenghuang Dancong are key representatives. Black tea, originating in China’s Fujian Province during the Ming Dynasty, is defined by full fermentation. The process comprising withering, rolling, fermentation, and drying facilitates the oxidation of polyphenols, leading to the formation of characteristic pigments like theaflavins and thearubigins. This results in a distinctive red liquor, a mellow flavor, and often sweet, floral, or fruity aromatic notes. Notable varieties include Dianhong, Keemun, and Lapsang Souchong. Dark tea undergoes a unique post-fermentation process. Key processing steps include fixing, rolling, pile-fermentation, and drying. This process imparts characteristic woody, aged, or Juhua aromas. Historically, these distinctive properties made dark tea a staple commodity along the ancient Tea-Horse Road, facilitating trade and socio-cultural exchange between China and neighboring regions. Prominent examples include Pu-erh, Fu brick tea, and Liubao tea.

Beyond the six major categories of tea derived from *Camellia sinensis* (L.) O. Kuntze and *Camellia sinensis* var. *assamica* (Masters) Kitamura, some countries and regions consume herbal teas produced from alternative botanical sources, with representative examples including Greek Mountain tea (*Sideritis* spp.), Mate tea (*Ilex paraguariensis* A. St.-Hil.), Rooibos tea (*Aspalathus linearis* (Burm. f.) R. Dahlgren), and Kuding tea (Ilex species such as *Ilex kudingcha* C.J. Tseng or *I. latifolia* Thunb.). Global tea production exhibits high geographic concentration, with China, India, Kenya, and Sri Lanka constituting the four dominant producing nations. As the center of origin for *C. sinensis*, China leads globally in total output, domestic consumption volume, and diversity of tea varieties. In contrast, India, Kenya, and Sri Lanka function as export-oriented producers where black tea dominates production and is extensively exported to European and North American markets. China’s export portfolio features specialty teas, including green, oolong, and Pu-erh teas, while its substantial domestic market absorbs the majority of production output.

### 2.2. Tea Aroma Characteristics

**Figure 2:**
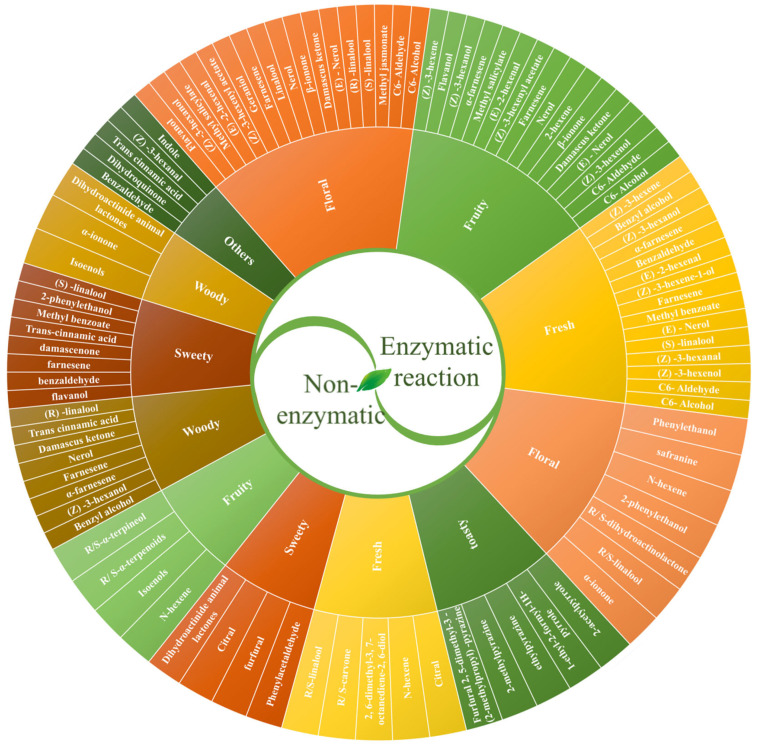
An aroma wheel is formed under enzymatic and non-enzymatic reactions. Note: Enzymatic and non-enzymatic reactions are two crucial processes in the formation of tea aroma. This figure illustrates the sensory aromas and their corresponding VOCs generated by these two types of reactions.

**Figure 3:**
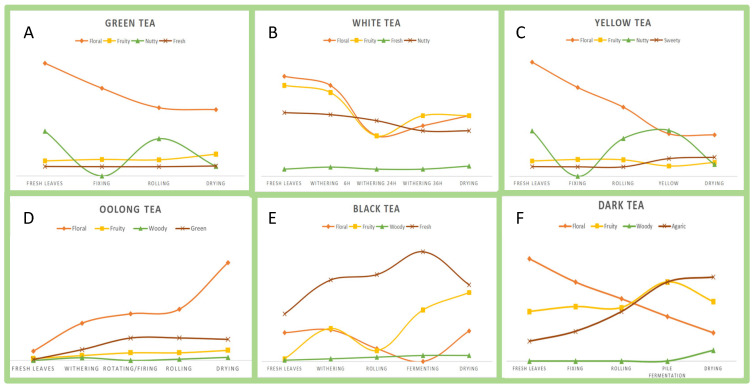
The changing trend of the aroma of six kinds of tea in different processing. Note: To investigate the variation trends of aroma compounds during the processing of the six major tea categories, this study selected a key representative compound for each aroma type. The content data of these compounds at different processing stages were obtained through a literature review, and the variation trend graphs were drawn accordingly. Each aroma type and its corresponding key compound are listed in the following text. (**A**) Floral—Linalool, Fruity—Heptanal, Nutty—Benzaldehyde, Fresh—Benzaldehyde [18,19]. (**B**) Floral—Linalyl acetate, Fruity—Phenylacetaldehyde, Fresh—(Z)-3-Hexen-1-ol, Nutty—Benzyl [20]. (**C**) Floral—Linalool, Fruity—Heptanal, Nutty—Benzaldehyde, Fresh—Benzaldehyde [18,21]. (**D**) Floral—Linalool, Fruity—Citral, Woody—Pinene, Green—3-Hexen-1-ol [22]. (**E**) Floral—Linalool, Fruity—Phenylacetaldehyde, Woody—Trans-linalool oxide (furanoid), Fresh—Methyl salicylate [23]. (**F**) Floral—Linalool, Fruity—Aldehydes, Woody—Guaiene, Agaric—1,2,3-Trimethoxybenzene [24].

Tea aroma is determined by the profile of VOCs, particularly aroma-active compounds, whose synthesis and release are importantly influenced during processing [13,14]. Tea leaves contain non-volatile precursors that generate aroma-forming VOCs. These VOCs primarily originate from four pathways: carotenoids as precursors, lipids as precursors, glycosides as precursors, and the Maillard reaction [15]. During processing, precursors predominantly undergo enzymatic and non-enzymatic conversion to release aroma-active compounds [16]. In Figure 2, we generate an aroma wheel to systematically categorize characteristic aroma attributes and their corresponding VOCs controlled by enzymatic and non-enzymatic reactions. Tea aroma is influenced by a range of factors, including tea cultivar, processing techniques, fresh leaf quality, harvesting season, and ecological and climatic conditions [17]. These factors collectively regulate the synthesis and abundance of aroma-active VOCs, thereby generating distinct aromatic profiles characteristic of different teas and underpinning the remarkable diversity observed in tea products. Notably, among these factors, the processing technique is the dominant influence on the final aroma profile formed. Fresh leaves undergo dehydration, mechanical stress, temperature variations, and microbial activity. These conditions induce dynamic changes in the abundance of key VOCs (Figure 3).

In Table 1, we also summarized the key aroma-active compounds identified from the six major tea types. The significant differences in aroma profiles show that comparing aromas provides a reliable basis for classifying teas.

Green tea, recognized as one of the earliest tea categories originating in China, preserves natural phytochemical constituents such as polyphenols, caffeine, and vitamins through its slight oxidation processing. These preserved compounds synergistically contribute to its signature sensory profile, dominated by floral, fresh, and chestnut-like aromas. It has been universally regarded as the organoleptic quality of premium green tea varieties. Nowadays, the aroma compounds have been well identified from different green teas, and the compounds including theaspirane, linalool, D-limonene, 3,7-dimethyl-1,5,7-octatrien-3-ol, 3-methylbutane, (E)-*β*-ionone, τ-cadinol, 3-methylbutanal, and 1,1,5-trimethyl-1,2-dihydronaphthalene are validated to play a crucial role in green tea aroma. Floral aromas primarily derive from volatile compounds such as linalool, geraniol, 1-hexanol, linalool oxides (furanoids), (E)-*β*-ionone, isoamyl acetate, and 2-methylpropanal, whereas chestnut-like aromas are attributed to 3-methylbutanal, indole, 2-ethyl-1-hexanol, *β*-damascenone, cedrol, cis-3-hexenol, linalool, hexanal, and 2-pentylfuran (Table 1) [25,26]. The fixation process critically determines green tea’s aroma profile. In this step, the high-temperature treatment (>180 °C) is used to inactivate enzymes, remove moisture, reduce grassy odors, and stimulate degradation of carotenoids and fatty acids aroma precursors into fragrance compounds, including geranylacetone, (Z)-jasmone, nerolidol, heptanal, and decanal [27]. Although these aroma compounds undergo significant transformations during processing, their metabolic pathways remain unclear. Green tea is further classified into four types based on fixation or drying methods: pan-fired, sun-dried, baked-dried, and steamed green teas. Each type of green tea exhibits unique aroma profiles: pan-fired tea features chestnut and roasted aromas, sun-dried tea exhibits solar-induced hay-like aroma, baked-dried tea emphasizes fresh and floral aromas, and steamed tea retains a pronounced fresh aroma [28]. Different drying methods critically shape green tea aroma. Pan-fired generates more volatiles than hot-air dried, promoting chestnut-roasted notes. Sun dried degrades lipids and carotenoids into aldehydes and ketones, yielding its faint scent [18,29].

White tea is characterized by fresh, floral, fruity, and sweet aroma. Its natural composition and health-promoting properties, particularly the potent antioxidant activity of flavonoid compounds, are widely recognized for their efficacy in scavenging free radicals and mitigating oxidative cellular damage [30]. It has been well found that the main volatile compounds of white tea are ethyl acetate, hexanal, linalool, dimethyl sulfide, 2-methylbutanal, phenylethanol, benzaldehyde, *β*-laurene, phenylacetaldehyde, (Z)-3-hexen-1-ol, 1-hexanol, (E)-2-hexenal, and geraniol [20,31]. Among these compounds, terpenoids geraniol, *α*-citral, and fatty acid-degraded (E, E)-3,5-octadien-2-one, (3E)-5-ethyl-6-methyl-3-hepten-2-one, cis-jasmone, hexanal, 3-octenal, phenylethylalcohol, benzaldehyde, γ-nonanolactone, and trans *β*-violets contribute to the fruity and sweet aromas [32]. Specifically, benzene acetaldehyde contributes a honey-like aroma. Methyl salicylate has a fresh and minty scent. Cis-linalool oxides (furanoid and pyranoid forms) impart a floral and earthy flavor, while linalool contributes a dominant bergamot aroma with secondary floral freshness [33]. Therefore, these compounds define the distinctive aroma of white tea. During withering, fresh tea leaves undergo water loss, biochemical transformations, and enzymatic oxidation reactions. This process constitutes the critical stage for white tea aroma development.

Yellow tea, a unique tea category in China, undergoes a distinctive yellowing process that drives substantial chemical transformations (Figure 1). This process not only endows yellow tea with characteristic flavors but also forms a complex aromatic bouquet dominated by potpourri-like and corn-like, complemented by nuanced floral, fruity, and sweet notes [34]. The key aroma compounds include (E)-*β*-ionone, (E, E)-2,4-heptadienal, *β*-selinene, *β*-cyclocitral, 2,3-diethyl-5-methylpyrazine, 2-ethyl-3,5-dimethylpyrazine, 2,5-dimethyl-3-(2-methylpropyl)pyrazine, 3,5-diethyl-2-methylpyrazine, (E, E)-2,4-decadienal, and related volatiles (Table 1) [35]. The potpourri-like and corn-like aroma mainly comes from 2-pyrrole formaldehyde, 3-ethyl-2,5-dimethylpyrazine, 2-ethyl-5-methylpyrazine, and 2,3-diethyl-5-methylpyrazine. The floral, fruity, and sweet aromas in young yellow tea and yellow bud tea mainly derive from phenylacetaldehyde, ethyl hexanoate, benzyl alcohol, geraniol, citral, neuraldehyde, and lauric acid [36]. The yellowing process leverages controlled thermo-humidification and enzymatic activity to induce metabolic shifts. During this process, ester catechins are oxidized and transformed under humid heat conditions. This oxidation reduces the content of ester catechins, thereby diminishing the astringency of the tea [37]. Additionally, Maillard reactions between amino acids and polyphenols generate sweet aromatic compounds, enhancing umami freshness while suppressing grassy notes [38]. This dual-pathway transformation fundamentally dictates yellow tea’s characteristic sensory profile, resulting in a balance between reduced astringency and enhanced sweet-umami complexity.

Oolong tea is characterized by its distinctive floral, fruity, and fresh aromas, which have been extensively documented in the literature [39]. These organoleptic properties stem from unique processing techniques that strategically integrate methodologies from green and black tea production, thereby preserving rich phytochemical complexes defining their biochemical signature. It has been shown that the main aroma compounds include 6-methyl-5-hepten-2-one, linalool, (Z)-furyl linalool oxides, linalool pyran oxides, phenylacetaldehyde, phenylethanol, and (E)-isoeugenol [40]. The floral and fruity aroma usually comes from benzaldehyde, benzyl alcohol, phenylacetaldehyde, phenylethanol, jasmine lactone, geraniol, (E)-nerolidol, jasmine lactone, and indole [41]. The rotating process, including shaking and cooling, is the key to forming color, aroma, and flavor through mechanical stress and dehydration. During this stage, shaking disrupts the cellular organization of the tea leaf edges, while cooling promotes both chemical changes in the tea and the uniform redistribution of moisture. These two dynamically and statically coupled processes facilitate the formation of characteristic “green leaves with red edge” and drive the flavor development.

Black tea, one of the most widely consumed tea categories globally, contains elevated caffeine levels that stimulate the central nervous system, enhance alertness, and improve cognitive performance. The characteristics of sweet, floral, and fruity aroma profiles are derived from complex volatile compositions, with over 600 aroma compounds identified to date [42]. It has been well recognized that phenylacetaldehyde, phenylethanol, geraniol, linalool, benzyl alcohol, (E)-2-octenal, 1-octen-3-ol, trans-linalool oxides (furans), methyl salicylate, trans *β*-violets, and (Z)-jasmonone are key aroma-active constituents (Table 1) [14,43]. Floral notes primarily correlate with linalool oxides, nerol, and geraniol derivatives, while fruity nuances arise from methyl salicylate, 1-penten-3-ol, and γ-decalactone [13]. Fermentation is the key process to influencing aroma formation: mechanical kneading destroys the cellular structure and facilitates the enzymatic oxidation of tea polyphenols, which in turn increases the aroma of tea. During the fermentation process, the aroma of tea gradually changes from a strong grassy odor to a sweet, floral, or fruity aroma, and the control of the fermentation degree is one of the keys to forming different black tea aromas. Biochemically, the characteristic aroma of black tea is predominantly derived from a series of interconnected processes, including the degradation of carotenoids, hydrolysis of fatty acids and glycosides, as well as the degradation of amino acids during fermentation [44]. These synergistic transformations during fermentation are fundamental in shaping the tea’s distinctive sensory profile and associated physiological effects. These enzymatic and oxidative cascades precisely convert primary metabolites into diverse volatile compounds, which directly shape the characteristic sensory profile of black tea and enhance its consumer acceptance.

Dark tea historically emerged from practical needs during early tea transportation and is closely linked to green tea. During previous long-distance transportation, green tea has been exposed to various natural environmental factors, including wind, sunlight exposure, and humidity fluctuations. These factors collectively induce a spontaneous and slow fermentation process. The historical origin of dark tea can be traced back to serendipitous events that occurred during transportation, and these occasional circumstances have also given rise to the unique aroma and flavor profile of dark tea. As a result, dark tea develops its unique quality characteristics, such as aged, betel nut-like, and Junhua aromas [45]. The aged aroma primarily originates from volatile compounds including 2-ethylfuran, 1,2-dimethoxybenzene, *β*-cyclocitral, 4-ethyl-1,2-dimethoxybenzene, 2-pentylfuran, and dihydro-*β*-ionone, which contribute to woody, stale, and roasted notes [46]. The betel nut-like aroma in Liubao tea arises from 2-methylnaphthalene, 1-methylnaphthalene, 2-heptanone, pulegone, and naphthalene, which impart minty, pungent, and smoky qualities. Conversely, the Junhua aroma in Fu brick tea stems from methyl salicylate, phenylethanal, cedrol, borneol, linalool, benzaldehyde, cis-linalool oxide, *α*-terpineol, and trans-linalool oxide, which blend floral, minty, woody, and fruity characteristics [47]. These aroma profiles are predominantly shaped by the pile-fermentation process. The controlled temperature, humidity, and duration facilitate the transformation of aroma compounds and microbial activity, yielding distinctive aromas in dark tea [48].

**Table 1:** Key aroma compounds identified from six types of tea with descriptive information.

No.	Compound	Odor Descriptor ^a^	Class	CAS	OT (mg/L) ^b^	Key Aroma Compounds (OAV > 1) ^c^						REF
						GT	WT	YT	OT	BT	DT	
1	Acetic acid, 4-methylphenyl ester	narcissus, phenol, animalic (⁑)	Ester	140-39-6	0.025						+	[16]
2	Acetic acid, hexyl ester	fruity, green, apple, banana, sweet (⁑)	Ester	142-92-7	0.115						+	[16]
3	Acetophenone	must, flower, almond (#)	Ketone	98-86-2	0.065						+	[16]
4	Anethole	sweet, exotic, flowery, stewed (⁑)	Aromatics	104-46-1	0.015				+		+	[10,16]
5	Benzaldehyde	bitter almond-like (‡)	Aldehydes	100-52-7	0.35	+		+		+	+	[3,9,13,16,17]
6	Benzene, (isothiocyanatomethyl)-	mild, watercress, dusty, medicinal, horseradish, oily (⁑)	Sulfur compounds	622-78-6	0.0007						+	[16]
7	Benzene, 1,3-bis(1,1-dimethylethyl)-	–	Aromatics	1014-60-4	1.1						+	[16]
8	Benzene, 1-ethyl-3-methyl-	–	Aromatics	620-14-4	0.088						+	[16]
9	Benzeneacetaldehyde	hyacinth, honey, clover, hawthorne, cocoa, grapefruit, peanut, floral (†)	Aldehydes	122-78-1	3.33	+	+		+	+	+	[3,6,10,14,18]
10	Benzyl alcohol	bitter almond-like (‡)	Alcohols	100-51-6	0.62					+	+	[13]
11	Beta-Ocimene	citrus, herbal, sweet, woody, terpene, herb (†)	Monoterpene	3779-61-1	0.0187					+		[14]
12	Biphenyl	pungent, rose, green, geranium (⁑)	Aromatics	92-52-4	0.0033			+			+	[9,16]
13	Butanoic acid, 2-methyl-, 2-methylpropyl ester	sweet, fruity (⁑)	Ester	2445-67-2	0.043						+	[16]
14	Butanoic acid, 3-methyl-, propyl ester	bitter, sweet, apple, fruity (⁑)	Ester	557-00-6	0.0087						+	[16]
15	Butanoic acid, butyl ester	fruity, banana, pineapple, green, cherry, tropical fruit, ripe fruit, juicy fruity (⁑)	Ester	109-21-7	0.028						+	[16]
16	Camphor	camphor (⁑)	Terpenoids	76-22-2	0.016						+	[16]
17	Carotol	pleasent, mild (⁑)	Terpenoids	465-28-1	0.008						+	[16]
18	Carvone	minty, licorice (⁑)	Terpenoids	99-49-0	0.067						+	[16,17]
19	Cedrol	wintergreen, woody (‡)	Alcohols	77-53-2	0.064	+					+	[3,17]
20	Cis-Linalool Oxide	flower, woody (#)	Heterocyclics	5989-33-3	0.006						+	[18]
21	Citronellol	floral, rose, lime (⁑)	Terpenoids	106-22-9	0.04						+	[16]
22	Coumarin	sweet, bitter almond-like (‡)	Heterocyclics	91-64-5	4.6			+				[9]
23	Decanal	citrus, soap, orange peel, tallow, waxy, floral, sweet, aldehydic (†)	Aldehydes	112-31-2	0.0001	+	+	+			+	[3,16]
24	Dihydroactinidiolide	fruity (‡)	Esters	17,092-92-1	0.0021	+						[1,9,17]
25	Dimethyl sulfide	corn-like (‡)	Sulfur compounds	75-18-3	0.0003	+				+		[1,2,15]
26	Dodecane	alkane (†)	Hydrocarbons	112-40-3	0.04		+				+	[5,16]
27	Ethanone, 1-(2-aminophenyl)-	grape, sweet (⁑)	Ketone	551-93-9	0.00027						+	[16]
28	Ethylbenzene	flower (#)	Aromatics	100-41-4	0.029	+						[3]
29	Eucalyptol	eucalyptus, herbal, camphor, medicinal (⁑)	Terpenoids	470-82-6	0.015						+	[16]
30	Eugenol	clove-like (‡)	Phenols	97-53-0	0.0025					+	+	[13,16]
31	Furan, 2-pentyl-	fruity, green, earthy, beany, vegetable, metallic (⁑)	Heterocyclics	3777-69-3	0.006						+	[16]
32	Geraniol	rose-like, citrus-like (‡)	Alcohols	106-24-1	0.797	+	+	+	+	+	+	[1,2,6,8,9,10,11,12,15,17]
33	Geranyl formate	fresh, rose, neroli, tea, rose, green (⁑)	Ester	105-86-2	0.2						+	[16]
34	Geranyl isobutyrate	sweet, floral, fruity, green, peach, apricot, rose (⁑)	Ester	2345-26-8	0.013						+	[16]
35	Geranylacetone	flowery, rose-like (‡)	Alcohols	3796-70-1	8.72						+	[17]
36	Heptanal	heavy, planty green odor, apricot-like, nutty flavor (†)	Ketone	111-71-7	0.003	+				+	+	[2,15]
37	Heptanoic acid, ethyl ester	fruit (#)	Esters	106-30-9	0.00116		+					[4]
38	Hexanal	grass, sweaty, tallow, fresh, fatty, fruity, aldehydic (#, †)	Aldehydes	66-25-1	0.005	+	+			+	+	[1,2,3,4,6,13,15,16]
39	Hexanoic acid	sweaty (‡)	Alkenes	142-62-1	0.673					+		[14]
40	Indole	mothball-like, flowery (‡)	Heterocyclics	120-72-9	0.04	+		+	+	+	+	[1,9,10,11,13,16]
41	Isoborneol	balsamic, camphor, herbal, woody (⁑)	Terpenoids	124-76-5	0.0085						+	[16]
42	Isobutyl isovalerate	sweet, fruity, apple, raspberry, green, banana (⁑)	Ester	589-59-3	0.034						+	[16]
43	Isopentyl hexanoate	fruity, banana, apple, pineapple, green (⁑)	Ester	2198-61-0	0.32						+	[16]
44	Jasmine lactone	coconut, sweet (‡)	Esters	25,524-95-2	1.65				+			[10]
45	Limonene	fresh orange, lemon-like (†)	Monoterpene	138-86-3	0.01		+					[5]
46	Linalool	citrus-like, flowery (‡)	Alcohols	78-70-6	0.0006	+	+	+	+	+	+	[2,3,5,6,7,8,9,10,11,12,13,14,15,18]
47	Methyl salicylate	minty (‡)	Esters	119-36-8	0.977	+	+	+	+	+	+	[1,3,4,5,9,10,12,13,14,15,17]
48	Naphthalene	strong mothball odor, dry, pungent, tarry (#, †, *)	Heterocyclics	91-20-3	0.05	+		+			+	[3,9,16]
49	Naphthalene, 1,2-dihydro-1,1,6-trimethyl-	licorice (⁑)	Aromatics	30,364-38-6	0.0025						+	[16]
50	Naphthalene, 1-methyl-	naphthyl, chemical, medicinal, camphor (⁑)	Aromatics	90-12-0	0.008						+	[16]
51	Naphthalene, 2,6-dimethyl-	grassy (⁑)	Aromatics	581-42-0	0.01						+	[16]
52	Naphthalene, 2-methyl-	sweet, floral, woody (⁑)	Aromatics	91-57-6	0.004						+	[16]
53	Nonanal	citrus-like, soapy (‡)	Aldehydes	124-19-6	0.001	+	+	+			+	[1,2,3,4,6,7,17]
54	Octanal	citrus-like, green (‡)	Aldehydes	124-13-0	0.0007	+	+					[1,3,4]
55	Octanoic acid, 3-methylbutyl ester	sweet, oily, fruity, green, soapy, pineapple, coconut (⁑)	Ester	2035-99-6	0.07						+	[16]
56	Octanoic acid, ethyl ester	fruit, fat (#)	Ester	106-32-1	0.01287		+					[4]
57	Pentanoic acid, 4-methyl-, ethyl ester	fruity (⁑)	Ester	25,415-67-2	0.006						+	[16]
58	Phenol, 4-propyl-	medicinal, phenol (⁑)	Phenol	645-56-7	0.107						+	[16]
59	Phenylethyl Alcohol	honey, rose, lilac (#)	Alcohols	60-12-8	0.399	+	+	+		+	+	[2,4,9,13,15]
60	Styrene	balsamic, gasoline (#)	Alkenes	100-42-5	0.000035			+				[9]
61	Trans-anethole	sweet, anisic, licorice, mimosa (⁑)	Aromatics	4180-23-8	0.057						+	[16]
62	Trans-Rose oxide	floral (⁑)	Terpenoids	876-18-6	0.0005						+	[16]
63	Trans-*β*-Ionone	violet-like, flower, raspberry-like aroma (†)	Ketone	79-77-6	<0.000001	+	+	+				[3,6,9]
64	Tridecane	alkane (†)	Hydrocarbons	629-50-5	0.042						+	[16]
65	Vanillin	vanilla-like, sweet (‡)	Aldehydes	121-33-5	0.053						+	[16]
66	*α*-Farnesene	fruity (‡)	Sulfur compounds	502-61-4	-						+	[17]
67	*α*-ionone	floral, sweet, woody, fruity, orris, raspberry, violet (†)	Ketone	127-41-3	0.0004		+	+	+	+	+	[4,9,11,13]
68	*β*-cyclocitral	mint, saffron, damascone, sweet, fruity, rose oxide (†)	Others	432-25-7	0.427				+		+	[10,11,17]
69	*β*-ionone	violet, orange, jam, seaweed, orris, raspberry, cedar wood odor (†, *)	Ketone	79-77-6	0.574	+		+	+		+	[1,8,10,11,15,17,18]
70	*β*-Myrcene	balsamic, must, spice (#)	Alkenes	123-35-3	0.013			+		+		[7]
71	*γ*-Nonalactone	coconut-like (‡)	Esters	104-61-0	0.03				+			[11]
72	(+)-Alpha-Pinene	–	Monoterpene	7785-70-8	0.0053					+		[14]
73	(*2E*,*4Z*)-2,4-Decadienal	fried, fatty, geranium, green, waxy (⁑)	Aldehyde	25,152-83-4	0.00007						+	[16]
74	(*E*)-2-Decenal	fatty, green (‡)	Aldehydes	3913-81-3	0.572				+		+	[10]
75	(*E*)-2-Hexenal	green apple-like (‡)	Aldehydes	6728-26-3	0.11					+		[13]
76	(*E*)-2-Nonenal	green, tallow-like (#)	Aldehyde	40,435-58-1	0.00008	+					+	[3]
77	(*E*)-2-Octenal	fatty, nutty (‡)	Aldehydes	2548-87-0	0.025	+	+				+	[3,4]
78	(*E*)-2-Pentenal	fatty, fruity (‡)	Aldehydes	1576-87-0	0.005				+			[10]
79	(*E*)-3-Penten-2-one	Fruity (#)	Ketone	4549-33-3	0.0015	+						[3]
80	(*E*)-Nerolidol	flowery, woody (‡)	Alcohols	40,716-66-3	0.006				+		+	[3,10,11]
81	(*E*)-*β*-Ionone	flowery, violet-like (‡)	Alcohols	79-77-6	0.000036	+		+		+		[2,7,15]
82	(*E*, *E*)-2,4-Heptadienal	fatty, flowery (‡)	Aldehydes	4313-03-5	0.000032	+		+			+	[1,8,17]
83	(*E*, *E*)-3,5-Octadien-2-One	creamy, fruity (#)	Ketone	1569-08-4	0.0005	+						[3]
84	(*Z*)-3-Hexen-1-ol	green, grassy (‡)	Alcohols	928-96-1	0.0039	+	+	+		+		[1,5,9,13]
85	(*Z*)-Hex-3-En-1-Yl Hexanoate	tender, fresh, clean aroma (⁑)	Esters	74,298-89-8	0.016	+						[3]
86	(*Z*)-Jasmone	woody, flowery (‡)	Alcohols	488-10-8	0.00002			+		+		[9,13]
87	*α*-Ionone	sweet, woody, floral, violet, orris, tropical, fruity (⁑)	Terpenoids	127-41-3	0.00378						+	[16]
88	1,3-Benzodioxole, 4-methoxy-6-(2-propenyl)-	spicy, warm, balsamic, woody (⁑)	Aromatics	607-91-0	0.088						+	[16]
89	1,6,10-Dodecatrien-3-ol, 3,7,11-trimethyl-	floral, green, waxy, citrus, woody (⁑)	Terpenoids	7212-44-4	0.01						+	[16]
90	1-Decanol	fatty, waxy, floral, orange, sweet, watery (⁑)	Alcohol	112-30-1	0.023						+	[16]
91	1-hexanol	oil, alcoholic, ethereal, resin, sweet, fruity, flower, green (†)	Alcohols	111-27-3	0.0056		+			+		[4,13]
92	1-Methylnaphthalene	pungent, rancid (#)	Aromatics	90-12-0	0.00002	+						[3]
93	1-Nonanol	fresh, clean, fatty, floral, rose, orange, dusty, wet, oily (⁑)	Alcohol	143-08-8	0.0053						+	[16]
94	1-Octanol	fresh orange rose odor penetrating aromatic odor, chemical, metal, burnt (#, †)	Alcohol	111-87-5	0.022		+		+		+	[4,12,16]
95	1-Octen-3-Ol	mushroom, lavender, rose, hay (†)	Alcohols	3391-86-4	0.001		+	+		+	+	[5,6,9,15,17]
96	2(3H)-Furanone, 5-ethyldihydro-	sweet, caramel (⁑)	Ester	695-06-7	0.26						+	[16]
97	2(4H)-Benzofuranone, 5,6,7,7a-tetrahydro-4,4,7a-trimethyl-, (*R*)-	musky, coumarin (⁑)	Heterocyclic compound	17,092-92-1	0.0021						+	[16]
98	2,4-Decadienal, (*E*, *E*)-	dusty, waxy, oily, soapy (⁑)	Aldehyde	25,152-84-5	0.00007						+	[16]
99	2,4-Di-tert-butylphenol	Phenol (⁑)	Phenol	96-76-4	0.5						+	[16]
100	2,6-Nonadienal, (*E*, *E*)-	fresh, citrus, green, cucumber, melon (⁑)	Aldehyde	17,587-33-6	0.0005						+	[16]
101	2,6-Nonadienal, (*E*, *Z*)-	cucumber, green (⁑)	Aldehyde	557-48-2	0.00001						+	[16]
102	2-Acetylthiazole	nutty, popcorn, roasted, peanut, hazelnut (⁑)	Heterocyclic compound	24,295-03-2	0.004						+	[16]
103	2-Cyclopenten-1-one, 2-hydroxy-3,4-dimethyl-	strong, caramel (⁑)	Ketone	21,835-00-7	0.02						+	[16]
104	2-Decenal, (*Z*)-	Tallow (⁑)	Aldehyde	2497-25-8	0.05						+	[16]
105	2-Dodecenal, (*E*)-	citrus, metallic, mandarin, orange, waxy, aldehydic (⁑)	Aldehyde	20,407-84-5	0.0073						+	[16]
106	2-hexenal	apple, cheesy, vegetable, banana, rancid, fatty, plum, almond (#, †, *)	Aldehydes	6728-26-3	0.00527					+	+	[14,17]
107	2-Methylbutanal	Malty (‡)	Aldehydes	96-17-3	0.0015	+						[1]
108	2-methylisoborneol	must, earth (†)	Alcohols	2371-42-8	0.000036					+		[14]
109	2-Methylpropanal	malty, pungent (‡)	Aldehydes	78-84-2	0.00049	+						[1]
110	2-Nonenal, (*Z*)-	orris, fatty, waxy, cucumber (⁑)	Aldehyde	60,784-31-8	0.0045						+	[16]
111	2-Octen-1-ol, (*E*)-	green, citrus, vegetable, fatty (⁑)	Alcohol	18,409-17-1	0.02						+	[16]
112	2-Pentylfuran	bean-like, fruity, green, earthy and vegetable-like smell (†)	Furan	3777-69-3	0.006	+					+	[3]
113	2-Propenal, 3-phenyl-	sweet, spicy, aldehydic, aromatic, balsamic, cinnamyl, resinous, honey, powdery (⁑)	Aldehyde	104-55-2	0.024						+	[14,16]
114	3-Mercapto-3-methylbutyl formate (ester)	sulfury, catty, caramel, onion, roasted coffee, roasted meat, tropical (⁑)	Ester	50,746-10-6	0.000002						+	[16]
115	3-Mercaptohexanol	sulfury, fruity, tropical (⁑)	Alcohol	51,755-83-0	0.00006						+	[16]
116	3-Methylbutanal	malty (‡)	Aldehydes	590-86-3	0.0005	+						[1,3]
117	3-Octanol	earthy, mushroom, herbal, melon, citrus, woody, spicy, minty (⁑)	Alcohol	589-98-0	0.078						+	[16]
118	3-Octanone	fresh, herbal, lavender, sweet, mushroom (⁑)	Ketone	106-68-3	0.0013						+	[16]
119	4-Decenoic acid, methyl ester, *Z*-	fruity, pear, mango, fishy, peach skin, green (⁑)	Ester	7367-83-1	0.003						+	[16]
120	4-Ethyl-1,2-Dimethoxybenzene	Mature (†)	Ethers	5888-51-7	0.0034						+	[18]
121	4-Phenyl-2-butanol	floral, peony, foliage, sweet, mimosa, heliotrope (⁑)	Alcohol	2344-70-9	0.0043						+	[16]
122	5-Methyl-2-thiophenecarboxaldehyde	sweet, almond, cherry, furfural, woody, acetophenone (⁑)	Heterocyclic compound	13,679-70-4	0.001						+	[16]
123	6-Methyl-5-hepten-2-one	fruity, nutty (‡)	Alcohols	110-93-0	0.656	+			+	+		[1,10,15]
124	6-Nonenal, (*E*)-	–	Aldehyde	2277-20-5	0.000022						+	[16]
125	6-Octen-1-ol, 3,7-dimethyl-, (*R*)-	citronella oil, rose, leafy, oily, petal (⁑)	Terpenoids	1117-61-9	0.04						+	[16]

^a^ Odor description found in the literature and website; #, http://www.flavornet.org/flavornet.html (accessed on 10 April 2025); †, https://cosylab.iiitd.edu.in/flavordb/search (accessed on 10 April 2025); *, https://pubchem.ncbi.nlm.nih.gov/ (accessed on 10 April 2025); ⁑, http://www.thegoodscentscompany.com (accessed on 10 April 2025); ‡, [43] ^b^ Odor thresholds were obtained from the following cited references and The Good Scents Company. +: OAV > 1, indicating they are key odorants. –: not retrieved ^c^ References: 1 [49], 2 [50], 3 [51], 4 [52], 5 [53], 6 [54], 7 [35], 8 [55], 9 [56], 10 [50], 11 [57], 12 [58], 13 [59], 14 [60], 15 [61], 16 [62], 17 [63], 18 [64]. The complete table is provided as Supplementary Materials in Table S1.

## 3. VOCs Extraction in Teas

Nowadays, the diversity of VOCs in tea is extensive. However, the identification of these VOCs is constrained by the limitations of extraction techniques. Therefore, the development of efficient and reliable VOCs extraction methods is crucial for further analysis. With the development of science and technology, contemporary analytical advances have facilitated the deployment of diversified extraction techniques. These methods not only improve the extraction efficiency but also facilitate a deeper understanding of the chemical composition and formation mechanisms underlying VOCs. In this study, five existing extraction methods were systematically assessed for the comparative analysis of advantages and limitations in capturing VOCs markers under standardized *Camellia sinensis* matrix conditions. They are simultaneous distillation extraction, solvent-assisted flavor evaporation, headspace analysis, supercritical fluid extraction, and solid-phase microextraction.

### 3.1. Simultaneous Distillation–Extraction

Simultaneous Distillation–Extraction (SDE), a technique integrating distillation and solvent extraction within a single apparatus, enabled the effective extraction of VOCs from teas and significantly advanced early-stage tea aroma research. Originally reported in 1964 [65], SED operates by continuously mixing sample vapors with solvent vapors in a sealed apparatus to achieve cyclic extraction. SDE achieves efficient concentration of VOCs by simultaneous steam distillation from both aqueous and organic phases. This method reduces solvent consumption and is particularly effective for extracting high-boiling-point substances, such as terpenoids in tea, while avoiding contamination of chromatographic systems by non-volatile lipids [66]. *β*-Ionone in black tea and pyrazines in dark tea require high-temperature distillation for effective extraction, so SDE is suitable for these compounds. Especially, SDE is characterized by low equipment costs and high reproducibility. Despite its advantages in high extraction efficiency, operational simplicity, and low sample requirements, the elevated operating temperatures (typically >60 °C) may induce secondary reactions. This may alter the composition of the extracted volatiles, resulting in deviations from the native aroma profile. To prevent burnt off-flavors from thermal decomposition of methy-cyclopentenolone, SDE is discouraged in yellow tea. Under the effect of heat, VOCs may undergo oxidation, degradation, etc., which in turn affects the identification of the final substance and generates off-flavors such as stale water and woody odor [66]. Recently, Cao et al. combined SDE with GC-MS to analyze Longjing tea samples with varying stale odor intensities, identifying hexanoic acid and trans-2-nonenal as key contributors to off-flavors [6]. However, a large number of VOCs have thermal instability, so SDE affects the authenticity of some compounds. In the subsequent research, SDE optimization should prioritize temperature modulation and cooling systems to minimize the impact on aroma compounds as much as possible.

### 3.2. Solvent-Assisted Flavor Evaporation

Solvent-assisted flavor evaporation (SAFE) combines two steps of solvent extraction and VOCs evaporation. The whole process is carried out at low temperatures (<50 °C, 10^−3^ mbar), which can extract and concentrate VOCs under mild conditions and minimize the loss of heat-sensitive components [67]. This method is highly effective for the comprehensive extraction of VOCs and is widely adopted in quantitative analyses. Particularly for white tea, yellow tea, and green tea, these tea types contain substantial compounds that require low-temperature extraction conditions. For instance, Qin et al. identified 173 volatile compounds in steamed green tea utilizing a combination of SAFE, headspace solid-phase microextraction, and GC-MS with two columns of different polarities [50]. Similarly, Feng et al. employed an integrated approach that combined SAFE with GC-MS, aiming to isolate and characterize volatile components while preserving their original odor profiles. This approach enables not only the comparison of aroma profiles across diverse tea varieties but also facilitates the sensory-relevant analysis of individual tea volatile compounds at the molecular level [68]. However, SAFE is hampered by labor-intensive procedures, low-temperature concentration protocols, and stringent vacuum maintenance. Additionally, polarity-selective compound loss is a significant drawback, primarily resulting from the inadequate coverage of the polarity spectrum by available solvent systems. Consequently, this limitation inherently restricts the range of compounds detectable by SAFE. Therefore, it is necessary to combine SAFE with other extraction techniques to extract more compounds.

### 3.3. Headspace Analysis

Headspace analysis (HS) is a technique for extracting VOCs in liquid or solid matrices. HS requires simplified sample pretreatment, incorporates straightforward operational protocols, and minimizes thermal degradation of aroma constituents. By directly capturing VOCs from tea headspace gas, HS eliminates solvent interference and enables rapid detection of low-boiling point aroma molecules. Due to the low volatility of certain compounds in black tea, solvent-assisted extraction is often necessary. Therefore, HS should be applied with caution. HS comprises two modes: static headspace (SHS) and dynamic headspace (DHS) [69,70]. In SHS, the gas phase above the sample is directly analyzed, which closely simulates human olfactory perception. However, SHS suffers from limited enrichment capacity for low-abundance aroma compounds, consequently hampering the detection of trace-level volatiles. In contrast, DHS overcomes this limitation by utilizing polymeric sorbents to concentrate these compounds through adsorption, followed by thermal or solvent desorption for analysis. This enrichment process significantly enhances sensitivity for trace analytes compared to SHS. However, DHS is characterized by complex equipment, high costs, and an inability to completely avoid the oxidative loss of volatile components. HS is commonly coupled with Solid-phase microextraction (SPME) for enhanced sensitivity in VOCs studies. For instance, Lin et al. identified 29 characteristic volatile compounds in jasmine tea using HS-SPME-GC-MS [71]. Similarly, Zheng et al. utilized HS-SPME-GC-MS to comprehensively characterize volatile profiles across four distinct Phoenix Dancong oolong teas and identified 36 aroma-active compounds as the key olfactory signatures [72]. To address the challenges of the HS, it is necessary to prioritize the development of miniaturized HS devices with integrated in situ trapping/desorption units to reduce volatile depletion, alongside the engineering of advanced sorbent materials for the selective enrichment of trace active compounds. This integrated approach can enhance the detection sensitivity for these key compounds. Additionally, implementing online HS-coupled sensor systems will enable real-time aroma quality monitoring during certain tea processing stages, thereby transforming analytical capabilities from laboratory characterization to industrial process control.

### 3.4. Supercritical Fluid Extraction

Supercritical fluid extraction (SFE) is a separation technique that utilizes the unique solvent properties of fluids above their critical pressure and temperature [73]. This method is characterized by non-flammability, low toxicity, environmental compatibility, and solvent recyclability. SFE predominantly employs supercritical CO_2_ (scCO_2_) as the extraction medium due to its chemical inertness, non-toxic nature, and facile separation, making it particularly suitable for extracting thermolabile VOCs from teas while preserving structural integrity through low-temperature operation [43]. The technique leverages scCO_2_’s high diffusivity and tunable solvent strength to selectively isolate target constituents without residual solvents, ensuring environmental safety [74]. Pressure and temperature modulation further enable targeted extraction of compounds with varying polarities. Nowadays, SFE serves as the widely used extraction method for both non-volatile and volatile compounds. For example, Ferrer et al. employed SFE to isolate polyphenolic fractions from oolong tea, subsequently evaluating their modulatory effects on dysregulated oncogenic pathways in breast carcinoma models [75]. Joshi et al. employed SFE to isolate aromatic components from Kangra orthodox black tea, subsequently performing aroma extract dilution analysis, which identified 11 aroma-active compounds [43]. The study further characterized the aroma qualities of these VOCs through systematic sensory evaluation. However, SFE’s adoption in VOCs extraction remains constrained by high-pressure infrastructure requirements, significant capital investment, and limited efficiency for polar molecules. Although polar entrainers (e.g., 5–15% ethanol) enhance polarity range, they may generate impurities that reduce extract purity. Therefore, future research should be used to combine these entrainers with strategies to minimize impurities. Additionally, developing scalable reactor designs will be crucial to fully exploit the potential of SFE for profiling the aroma of teas.

### 3.5. Solid-Phase Microextraction

Solid-phase microextraction (SPME) is a solvent-free and environmentally friendly sample preparation technique, first reported by Arthur and Pawliszyn in 1990 [76]. This method employs fiber probes coated with porous polymeric sorbents. Compared to conventional methods, SPME offers advantages such as operational simplicity, rapid extraction, and elimination of solvent interference, enabling accurate replication of tea aroma profiles. After that, SPME has been widely used in various types of VOCs extraction. For instance, Baptista et al. employed SPME-GC to comparatively analyze VOCs across green tea varieties [77], while Wang et al. applied this method to characterize the key VOCs in Pu-erh tea through advanced SPME-GC-O/MS [78]. Recently, SPME coupled with HS has become a mainstream approach in tea volatilities research, which significantly promotes the study of VOCs. Sun et al. conducted a systematic investigation using HS-SPME-GC-MS to monitor real-time VOCs profiles in green tea infusions throughout the brewing process. Their research developed a quantitative framework defining optimal steeping parameters, identifying critical temporal thresholds for aroma compound liberation, and establishing standardized quality control strategies [79]. However, SPME exhibits limited adsorption capacity for polar compounds and suffers from fiber-to-fiber reproducibility due to matrix interference from competing phenolic adsorbates. Sheibani employed both SDE and SPME to extract the oolong tea’s aroma compounds. Under identical conditions, SDE identified 128 volatile compounds, whereas SPME detected 59 volatile substances. The composition of the volatiles separated by the two methods varied significantly, yet they provided complementary information [80].

As mentioned above, there is no universally optimal extraction method for tea aroma extraction. Developing high-purity, eco-friendly, and cost-effective extraction techniques is critical for advancing tea aroma studies. Effective extraction of tea aroma compounds demands method customization aligned with specific tea varieties. Nevertheless, the inherent instability and potential toxicity of these volatile constituents present universal challenges across all methodologies. Instability originates from the compounds’ pronounced reactivity, which facilitates their decomposition through oxidation or thermal degradation during extraction, consequently altering the tea’s aromatic profile [81]. Toxicity concerns arise from two primary sources. On the one hand, certain VOCs possess intrinsic toxic properties, as exemplified by benzaldehyde and methyleugenol. Although their concentrations in tea matrices typically remain below hazardous thresholds, stringent prevention of chemical reagent residues is essential. On the other hand, toxicity may emerge when unstable compounds undergo degradation via oxidative or thermal pathways in solvent systems. Mitigation requires integrated strategies: optimization of extraction parameters through judicious solvent selection and temperature-pressure regulation; adoption of green solvents to reduce chemical burden; and execution of comprehensive safety assessments. These measures collectively ensure compound integrity and safety throughout extraction, storage, and application sequences.

## 4. VOCs Analysis in Teas

**Figure 4:**
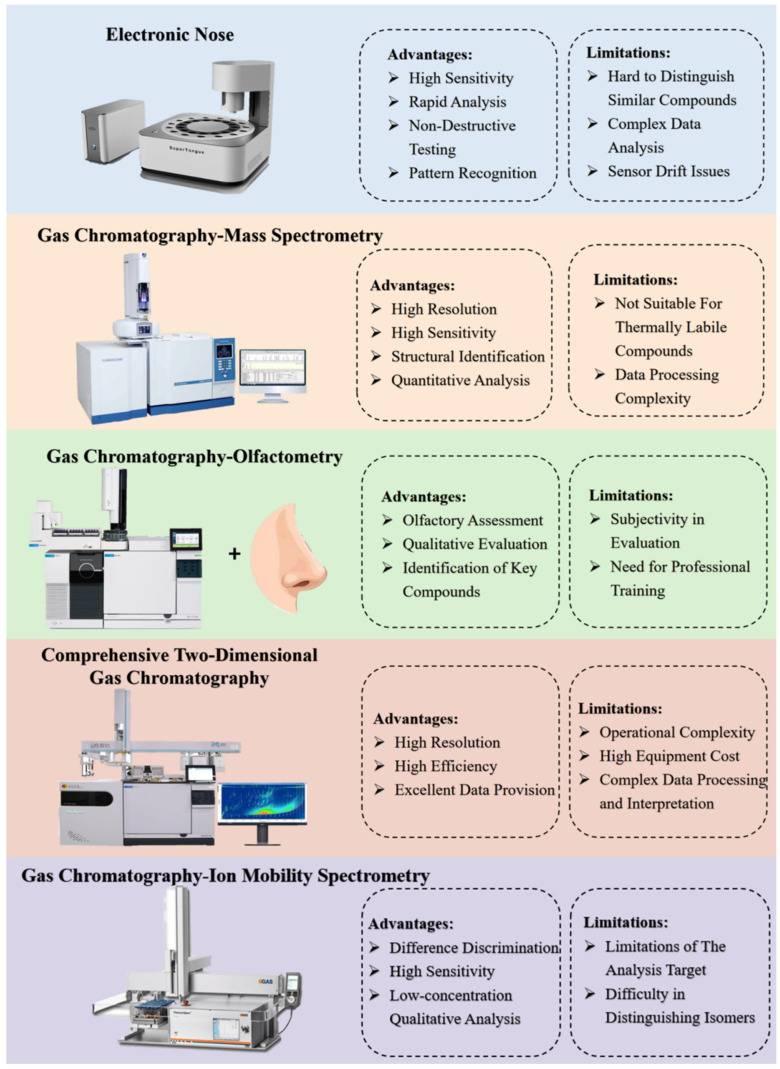
Advantages and disadvantages of different aroma identification techniques.

A comprehensive analysis of VOCs is essential for assessing tea quality attributes and investigating their aroma formation mechanisms. This analytical process underpins critical research domains including quality assessment, varietal discrimination, process optimization, flavor innovation, and quality standardization. It not only provides an indispensable tool for quality control and scientific research for tea but also offers essential technical support for the development of the tea industry. The commonly employed techniques for detecting these compounds have been widely used in tea research and include electronic nose (E-nose), gas chromatography-mass spectrometry (GC-MS), gas chromatography-olfactometry (GC-O), comprehensive two-dimensional gas chromatography (GC × GC), and gas chromatography-ion mobility spectrometry (GC-IMS). This review has provided a detailed overview of the applications of these five technologies in the field of tea aroma analysis, and has analyzed their respective strengths and limitations in Figure 4.

### 4.1. E-Nose in VOCs Analysis

The E-nose mimics mammalian olfaction through cross-reactive sensor arrays coupled with multivariate pattern recognition algorithms to detect and classify VOCs [82,83]. This technology integrates sensor arrays, signal processing circuits, data analysis software, and artificial intelligence algorithms, enabling applications in odor profiling, food quality assessment, environmental monitoring, medical diagnostics, and explosive detection [84,85,86]. In tea aroma studies, E-nose has been instrumental in rapid aroma differentiation. It exhibits high sensitivity, rapid analysis, and non-destructive testing capabilities, as well as robust pattern recognition for classifying complex odor profiles. For instance, Yang et al. employed E-nose systems to evaluate the unique flavor profiles of Japanese green teas [87], while Qin et al. combined E-nose with GC-MS to compare flavor compounds across green and black tea grades [88]. More recently, Zhang et al. utilized E-nose to analyze flavor and aroma variations in asparagus tea under blanched and unblanched conditions [89]. The E-nose offers significant advantages. These include high sensitivity to trace volatile compounds, rapid analysis enabling real-time monitoring, non-destructive testing that preserves sample integrity, and advanced pattern recognition for complex aroma profiling. However, the E-nose detects VOC fingerprints reflecting overall composition, but cannot identify individual compounds. This limitation occurs because its sensor array responds to collective VOC properties, not single compounds. Structural elucidation, thus, requires hyphenated techniques like GC-MS for specific VOC identification. Additionally, the technology faces limitations: difficulty in distinguishing structurally similar compounds; reliance on complex multivariate data analysis; and susceptibility to sensor drift, particularly under high humidity (>70% RH) and temperature (>35 °C). This drift requires frequent calibration and poses operational challenges.

### 4.2. GC-MS in VOCs Analysis

GC-MS is an analytical instrument integrating gas chromatography (GC) and mass spectrometry (MS). GC, a separation technique, leverages differences in partitioning coefficients between a mobile phase (typically inert gases like helium or nitrogen) and a stationary phase (liquid/solid coatings within the column) to resolve sample components. As analytes traverse the column, they elute at distinct retention times, generating characteristic chromatographic peaks [90]. However, MS identifies molecules through mass-to-charge ratio (*m*/*z*) measurement and fragmentation patterns. In GC-MS systems, the GC effluent is directly interfaced with the MS ion source, enabling real-time analysis of separated compounds. Modern advancements include triple quadrupole MS (QqQ MS), enabling femtomole-level detection via multiple reaction monitoring (MRM) [91] alongside high-resolution time-of-flight MS (TOF MS) with sub-5 ppm mass accuracy for untargeted omics [92]. As the central technique for VOCs research, GC-MS enables high resolution, sensitive identification, and quantification of thousands of compounds in complex matrices, such as food flavors and aromatic profiles [93]. Guo et al. employed GC-MS to characterize the dynamic changes in aroma profiles throughout the processing of large-leaf yellow tea. The analysis revealed distinct differences in VOCs between processing stages, identifying a total of 178 volatiles [94]. However, its efficacy depends on compound volatility, thermal stability, and instrumental configurations. While high-resolution systems like GC-TOF MS and GC-QqQ MS enhance trace compound detection, their advanced hardware requirements significantly elevate operational costs. Additionally, GC-MS is unsuitable for thermally labile compounds and demands extensive data preprocessing to extract actionable insights.

### 4.3. GC-O in VOCs Analysis

GC-O integrates chromatographic separation with human olfactometric detection to characterize aroma-active compounds. This technique resolves complex volatile mixtures into individual components, enabling qualitative odor profiling of each eluted analyte. GC-O facilitates the construction of aroma databases by cataloging odorant profiles of foods, serving as a critical reference in flavor chemistry [95]. For instance, Ma et al. identified 52 aroma-active compounds in Dianhong black tea using GC-O combined with aroma extract dilution analysis (AEDA) and odor-specific magnitude estimation (OSME) [96]. As a core methodology, GC-O is further enhanced when paired with odor activity value (OAV) analysis to pinpoint key odorants. By calculating the ratio of a compound concentration to its odor threshold (the minimum concentration perceptible to humans), OAV quantifies the contribution of individual compounds to the overall aroma profile. For example, Tian et al. demonstrated *β*-damascenone as the dominant volatile in aged Pu-erh tea via OAV [97]. And Wang et al. employed response surface methodology (RSM) coupled with OAV to design the response variable for aroma sensory evaluation. This approach ultimately identified the optimal processing parameters for floral green tea [98]. GC-O directly links odor activity to chemical composition through sensory evaluation, thereby facilitating the qualitative identification of key aroma compounds. However, while sensory evaluation methods are essential for characterizing aroma perception, they remain susceptible to subjectivity from human variability. Thus, rigorous training and validation of sensory panels are required to enhance reliability. For precise aroma identification in tea research, GC-O is particularly effective for establishing sensory relevance. This technique directly couples GC separation with human sensory assessment to correlate chemical data with perception. Future research should prioritize integrating artificial intelligence with multi-omics data to establish predictive aroma profiling models, enabling objective and quantifiable aroma characterization that transcends subjective limitations.

### 4.4. GC × GC in VOCs Analysis

GC × GC integrates two chromatographic columns with distinct separation mechanisms. The first dimension column is designed to separate VOCs in the sample, while the second dimension column further resolves co-eluting compounds from the first dimension [99]. This dual-dimensional separation significantly enhances the separation efficiency and resolution of VOCs in complex samples. Guan et al. employed GC × GC-O-MS to assess volatile changes in fermented tea beverages, revealing that lactic acid bacteria fermentation-induced decarboxylation and saccharide metabolism, consequently increasing alcohols and ketones, which modulated the aroma profile [100]. With the integrated application and continuous evolution of GC × GC, comprehensive two-dimensional gas chromatography time-of-flight mass spectrometry (GC × GC-TOFMS) has garnered widespread acclaim. Kang et al. analyzed the aroma profiles of the world’s four most famous black teas using GC × GC-TOFMS and GC-O techniques [101]. Wang et al. detected 26 VOCs in Wuyi Rock Tea through GC × GC-TOFMS [102]. GC × GC provides exceptional resolution and peak capacity for separating complex mixtures, along with multidimensional data that improve compound identification. However, GC × GC technology faces significant challenges, including high operational complexity, substantial instrument costs, and demanding data interpretation requirements. To advance analytical workflows in multidimensional chromatography, it is critical to develop more user-friendly interfaces, robust automated method development routines, and simplified system configurations that reduce operational complexity while enhancing routine analysis. These improvements should be paired with sophisticated, intuitive, and automated software solutions specifically tailored for processing GC × GC data, including enhanced peak deconvolution algorithms, spectral matching libraries optimized for GC × GC-TOFMS, and efficient data compression techniques. By further integrating cheminformatics approaches, these innovations will collectively streamline workflows, accelerate compound identification and quantification, and ensure reliable analytical outcomes even for highly complex datasets. This dual focus on system accessibility and advanced computational capabilities bridges the gap between operator efficiency and cutting-edge analytical precision, positioning the technology for broader adoption across diverse laboratory environments.

### 4.5. GC-IMS in VOCs Analysis

GC-IMS consists of a gas chromatography system and an ion mobility tube, and it is characterized by high sensitivity and differential discrimination capabilities [103]. The synergy between the rapid analysis capabilities of GC and the heightened sensitivity of IMS positions GC-IMS as a pivotal tool for detecting and characterizing VOCs [104]. Recently, Guo et al. successfully characterized the aroma profiles of three types of oolong tea using GC-IMS, and 27 volatiles were identified [39], while Yan et al. applied GC-IMS to identify and quantify volatile compounds in Conggu black tea, highlighting its advantages of rapid detection, minimal sample preparation, and non-destructive nature [105]. The two-dimensional or three-dimensional spectra generated by GC-IMS can provide an intuitive visualization of differential patterns in VOCs. Combined with chemometric methods, GC-IMS can effectively decipher complex VOCS. However, a key limitation is the restricted number of compounds in GC-IMS databases, which hampers comprehensive VOCs identification. To address this challenge, Guo et al. proposed strategies and methods to improve the qualitative analysis of volatile compounds using GC-IMS and suggested potential applications in tea quality control, online tea production monitoring, and tea storage environment management [106].

### 4.6. Advanced Analysis in Aroma Identification

In recent years, advances in characterizing VOCs have revealed significant limitations of relying on single analytical methods to meet modern demands for comprehensive profiling. These limitations often include insufficient resolution for complex mixtures, limited compound identification specificity, or lack of holistic sensory information. Consequently, a growing number of studies now employ integrated analytical frameworks to comprehensively characterize VOCs, combining multiple identification techniques for enhanced accuracy and reliability. Integrated analytical frameworks combining complementary techniques have emerged as a powerful strategy to overcome these constraints and achieve a more complete characterization of VOCs.

For example, GC-MS is frequently used for VOCs’ identification and detection in foods due to its versatility and robust qualitative and quantitative capabilities. However, it requires complex sample preparation. Conversely, E-nose offers advantages in speed and cost-effectiveness for rapid fingerprinting but can suffer from limited stability and repeatability due to sensor drift [107]. To address the inherent limitations of each standalone technique, integrated approaches like GC-E-nose have been developed. GC-E-nose leverages the separation power of GC to reduce mixture complexity before the effluent is introduced into an E-nose sensor array. This coupling enhances the E-nose’s discrimination ability by minimizing interference and provides a hybrid dataset combining chromatographic separation with sensor-based pattern recognition. This integrated technology has demonstrated success in applications such as the rapid analysis and identification of Pu-erh tea [108], suggesting its potential for efficient screening and characterization of complex aromas. Such synergistic combinations exemplify the growing trend of utilizing comprehensive analytical platforms to unlock deeper insights into tea aroma chemistry.

Moreover, researchers increasingly combine complementary analytical techniques to comprehensively characterize VOCs. For instance, Huang et al. employed automated thermal desorption-gas chromatography-mass spectrometry (ATD-GC-MS) to characterize regional differences in the aroma compounds of Fuyun No. 6 black tea, revealing significant variations in volatile profiles that contribute to distinct sensory characteristics [109]. This synergistic application of multiple techniques significantly enhances the accuracy and sensitivity of volatile compound detection and identification. Importantly, this combined approach gives a fuller picture of complex aromas. It tackles the challenges arising from sample complexity and also helps bridge the gap between chemical composition and sensory perception, a key goal in modern aroma research.

**Figure 5:**
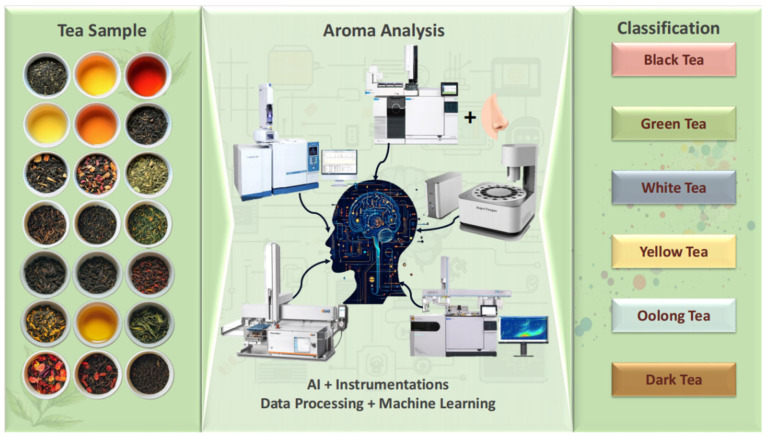
The combination of AI with aroma detection instruments for the identification of the teas. Note: AI can be integrated with various detection instruments, leveraging data processing and machine learning to perform tea classification. This approach involves collecting data from the instruments, processing it to extract meaningful features, and then applying machine learning algorithms to analyze and categorize the tea samples based on their unique characteristics. Refer to AI software: Google Cloud AI (version: 1.28.0) (https://cloud.google.com/ai, accessed on 10 April 2025), Azure Machine Learning Studio (version: azure-ai-ml 1.10.0) (https://azure.microsoft.com/en-us/products/machine-learning, accessed on 10 April 2025), DataRobot (version: 2024.04) (https://www.datarobot.com/, accessed on 10 April 2025).

Building upon these integrated analytical platforms, artificial intelligence (AI), particularly machine learning applied to data from spectroscopy, E-nose, GC-MS, and other sensors, has emerged as a transformative tool in tea aroma research. Xu et al. employed machine learning models, specifically principal component analysis-support vector machine (PCA-SVM) and principal component analysis-wavelet neural network (PCA-WNN), to accurately classify six key VOCs [110]. The approach of combining AI with VOCs detection instruments for the identification of teas is illustrated in Figure 5. Their proposed theoretical framework integrates AI with multiple detection techniques. Specifically, based on VOCs datasets obtained from GC-MS, their approach combines chemometric methods with machine learning algorithms to establish aroma fingerprints of tea samples. Critically, this process is validated through OAV analysis and aroma recombination experiments. The framework enables the direct identification of tea aroma types by calculating ratios of key aroma compounds, which significantly enhances the accuracy and scientific foundation of sensory evaluation. Furthermore, AI’s application extends beyond GC-MS integration. It can consolidate and model data from diverse analytical methods, presenting results in structured, quantifiable, and often predictive digital formats. This capability provides novel analytical perspectives and opens new research avenues for tea aroma characterization and related applications.

## 5. Application of Tea Aroma

Tea aroma, recognized for its natural and highly valued fragrance, is not only fundamental to the traditional tea experience but also exhibits significant potential for diverse applications across modern industries. The characteristic freshness and subtlety of tea aroma can impart a desirable natural sensory profile to products such as cosmetics, perfumes, personal care items, and all kinds of foods, enhancing their sensory appeal. Furthermore, the inherent complexity of tea aroma compounds contributes richness and depth to product fragrance formulations, enhancing their sensory appeal and marketability. Beyond their direct olfactory contribution, these VOCs may offer additional value through potential aromatherapeutic effects. Inhalation of tea aroma has been associated with mood modulation, such as promoting relaxation or reducing stress perception in some contexts [111]. This potential to influence well-being via the olfactory pathway adds another compelling dimension to the application of tea-derived fragrances, significantly broadening their appeal and scope in functional product development.

### 5.1. Essential Oil Application

Essential oils are well-known for their diverse medicinal properties, encompassing antimicrobial, anti-inflammatory, antioxidant, and analgesic effects. Building on this foundation, tea essential oils, produced by extracting VOCs from teas, present significant potential for innovative product development. Tea essential oils offer pleasant sensory profiles and exhibit stress-relieving and anxiolytic properties, leading to their wide application in daily care, cosmetics, massage therapy, and aromatherapy [112,113,114]. The therapeutic potential of tea essential oils is demonstrated through diverse clinical applications. Consequently, tea essential oils represent a valuable and versatile resource for creating multifunctional products across health and wellness. To fully realize this potential and ensure responsible utilization, future research should prioritize: (1) investigating the broader spectrum of therapeutic potentials of tea essential oils; (2) optimizing extraction methods to maximize yield and bioactive compound potency; and (3) conducting comprehensive safety and efficacy evaluations of tea essential oil products. These investigations are essential to support their widespread adoption in health, wellness, and other industries.

### 5.2. Food Additive Application

Tea-derived ingredients, encompassing both distinctive aroma compounds and bioactive non-volatile compounds, offer significant value in food applications. Beyond enhancing flavor, the incorporation of tea solids or extracts can improve the functional properties of foods. For instance, using matcha in cakes imparts a characteristic green tea flavor and significantly increases phenolic content and antioxidant capacity [115]. Similarly, adding green tea during beer brewing modifies the flavor profile and enhances antioxidant activity, which can help retard flavor deterioration [116]. Distinct from these functional contributions, the characteristic aroma compounds of tea are central to its flavor identity. Purified tea aroma extracts serve primarily as flavoring agents. They can impart an authentic tea-like aroma to foods, typically with negligible color contribution, making them ideal for applications where visual appearance is critical. An example is their use in drinks, where they enhance the aromatic profile and contribute to a perception of sweetness [117]. However, for optimal utilization of tea aroma extracts in complex food matrices, further research is needed to refine the targeted delivery and controlled release of specific key VOCs to achieve the desired sensory profile.

### 5.3. Daily Necessities Application

The application of tea aroma in fragranced daily products is experiencing significant growth. Beyond enriching the sensory dimension of consumer experiences, this trend presents substantial innovation opportunities for the fragrance industry. The use of tea fragrances has expanded from traditional items to various daily products, including perfumes, shampoos, body washes, and skin care products [118,119]. In perfume design, tea aroma can serve as the dominant accord or be artfully blended with complementary scents like floral or fruity notes to create unique olfactory compositions. In addition, products like soaps and skincare incorporating tea extracts deliver a distinctive tea fragrance and may offer additional functional benefits, such as antioxidant and antimicrobial properties derived from bioactive compounds like polyphenols, contributing to skin health. Tea-scented products offer benefits beyond their primary functions. For instance, tea-scented detergents effectively clean while imparting a refreshing aroma that enhances the laundry experience. Similarly, tea-scented candles create a warm ambiance in home settings and harness the aromatherapeutic potential of the released tea fragrance. Importantly, the inhalation of tea aroma, as a natural scent, has been associated with stress and anxiety relief, promoting an overall sense of well-being [120,121]. This essential benefit underpins its growing appeal in aromatherapy and health-centered product applications.

Driven by increasing consumer demand for natural and healthy products, the utilization of tea aroma in consumer goods is poised for significant growth and diversification. To capitalize on this trend and cater to diverse consumer preferences, future advancements will prioritize two key areas: (1) Enhanced scent customization leveraging novel delivery systems and formulation technologies to optimize tea aroma performance and create tailored experiences; and (2) Strengthened environmental sustainability across the supply chain, supported by advanced extraction methods. Achieving these goals necessitates leveraging modern technologies. Advanced extraction methods can improve efficiency and yield while minimizing environmental impact. Concurrently, novel delivery systems and formulation technologies can optimize the sensory performance of tea aroma in various products and enable tailored scent experiences.

## 6. Conclusions

In this review, we conducted a detailed analysis of the distinct aroma profiles and their corresponding VOCs across six major tea types. The methodologies, including extraction techniques (SDE, SAFE, HS, SFE, SPME) and analytical approaches (E-nose, GC-MS, GC-O, GC × GC, GC-IMS) used in VOCs analysis were systematically summarized. With the development of technology, the accuracy and efficiency of VOCs characterization are promoted, which provides solid technical support for revealing the formation of tea aroma quality. We also proposed the AI technique in tea aroma analysis area, which will provide a new way to conduct comprehensive and accurate tea aroma analysis. Furthermore, the potential applications of tea aroma in essential oils, food additives and daily products are also proposed. Importantly, this review will lay a solid foundation for further investigations in VOCs and tea aroma by technologies and provide significant insights into the development and application of tea fragrance for the tea industry.

## Data Availability

No new data were created or analyzed in this study.

## Supplementary Materials

The following supporting information can be downloaded at: https://www.mdpi.com/article/10.3390/foods14152574/s1, Table S1: Aroma compounds identified from six types of tea with descriptive information.

## Abbreviations

The following abbreviations are used in this manuscript:

VOC	Volatile Organic Compounds
OAV	Odor Activity Value
SDE	Simultaneous Distillation-Extraction
SAFE	Solvent-Assisted Flavor Evaporation
HSA	Headspace Analysis
SFE	Supercritical Fluid Extraction
SPME	Solid-Phase Microextraction
E-nose	Electronic Nose
GC-MS	Gas Chromatography-Mass Spectrometry
GC-O	Gas Chromatography-Olfactometry
GC × GC	Comprehensive Two-Dimensional Gas Chromatography
GC-IMS	Gas Chromatography-Ion Mobility Spectrometry
GC × GC-TOFMS	Comprehensive Two-Dimensional Gas Chromatography Time-Of-Flight Mass Spectrometry
ATD-GC-MS	Automated Thermal Desorption-Gas Chromatography-Mass Spectrometry
GC-QqQ MS	Gas Chromatography- quadrupole Mass Spectrometry
GC-TOF MS	Gas Chromatography-Time-of-Flight Mass Spectrometry
MALDI	Matrix-Assisted Laser Desorption/Ionization
ESI	Electrospray Ionization
BC	Breast Carcinoma
RSM	Response Surface Methodology
AI	Artificial Intelligence
PCA-SVM	Principal Component Analysis-Support Vector Machine
PCA-WNN	Principal Component Analysis-Wavelet Neural Network
